# Evaluation of the hemostatic efficacy and safety of an absorbable hemostatic powder based on a porcine intracranial hemorrhage model

**DOI:** 10.3389/fbioe.2026.1746208

**Published:** 2026-02-10

**Authors:** Jiali Shi, Yun Zhou, Yang Fu, Jin Wang

**Affiliations:** 1 Hangzhou Singclean Medical Products Co., Ltd., Hangzhou, China; 2 College of Marine Life Sciences, Ocean University of China, Qingdao, Shandong, China

**Keywords:** absorbable hemostatic powder, effectiveness, neurosurgery, porcine intracranial hemorrhage model, safety

## Abstract

**Introduction:**

This work aimed to evaluate the hemostatic efficacy and safety of an absorbable hemostatic powder in intracranial hemorrhage of porcine model.

**Method:**

Absorbable hemostatic powder was prepared from potato starch via mulsification and cross-linking technology, followed by structural characterization. An intracranial and extracranial injury hemorrhage in Bama miniature pigs’ model was established to evaluate the safety and effectiveness of the absorbable hemostatic powder. The biophysical characteristics of the absorbable hemostatic powder were compared with the control group and marketed product.

**Results:**

Absorbable hemostatic powder is a multi-microsphere with a spatial reticular microstructure with a porosity of 61.77%. Its water absorption rate reached over 1,200%. Animal research results showed that the hemostasis time and bleeding volume of the absorbable hemostatic powder were 73.50 ± 29.08 s and 3.90 ± 2.09 g, respectively. Compared with the model control group, both were significantly reduced (*P* < 0.01). Its hemostatic efficacy was comparable to that of the currently marketed product, but the required dosage was significantly lower (*P* < 0.01), being half that of the marketed products. The degradation study demonstrated that the absorbable hemostatic powder was almost completely degraded in the *in vitro* simulated environment within 48 h, and the degradation and absorption process was essentially completed after 42 days of implantation in the body. At the same time, no abnormalities were observed in the general signs, blood coagulation indicators, and pathological examination of the miniature pigs during the research process.

**Discussion:**

The conclusion indicated that the absorbable hemostatic powder was safe and effective in neurosurgical applications. Its porous structure design combines the advantages of rapid hemostasis and biocompatibility, providing a new idea for the development of clinical hemostatic materials.

## 1 Introduction

In neurosurgical clinical events, bleeding and related complications are particularly concerning issues for neurosurgeons. Careful hemostasis measures must be taken to control the bleeding especially during the operation. During craniotomy, extensive bleeding from multiple surface vessels such as the dura mater and the brain is extremely troublesome. Although bipolar electrocoagulation can be used to stop the bleeding in the surgical field (Soldozy et al., 2020), this method may have the potential risk of damaging normal nerve tissues. In some cases, it often leads to neurological dysfunction, and controlling exudative bleeding within a large surgical scope is also quite challenging. Therefore, external hemostatic agents have been widely used by neurosurgeons in clinical practice to achieve the goal of adequate hemostasis. However, some clinical practices have found that the application of local hemostatic agents may cause foreign body reactions, including mass effects and edema, and may also cause artifacts in the magnetic resonance imaging (MRI) examinations of patients undergoing tumor resection, thereby interfering with the examination results (Kothbauer et al., 2001; Zhang et al., 2024)^.^ Gelatin sponge, as a local hemostatic agent, is most commonly used in neurosurgical operations (Yao et al., 2013; Fiss et al., 2007). Because the absorbable gelatin sponge expands when absorbing blood and fluids, it may cause pressure or damage to the nerves or other important organs or tissues nearby. Moreover, the adhesion strength of the gelatin sponge is poor. It is prone to float and shift when there is a large amount of bleeding at the wound site, which affects the hemostasis effect. Additionally, the absorption time after surgery is long, which affects the imaging observation (Huo et al., 2012).

Faced with these challenges, exploring novel hemostatic materials has become a research focus. The porous powder-based hemostatic method has garnered significant attention due to its superior hemostatic efficacy and biocompatibility advantages. Among these materials, starch-based materials have emerged as an ideal choice owing to their natural degradability and low toxicity (Björses et al., 2011; Li et al., 2017). Physical and chemical techniques are employed to optimize the structure of starch-based materials, thereby enhancing the stability and adhesive strength of the hemostatic agent. The starch hemostatic material utilizes the surface micropores of the micro-polymerized hydrocolloid (MPH) particles, which are similar to a filtering sieve with varying pore sizes. These pores can rapidly absorb water and other components such as platelets and blood cells from the blood within a short period of time. These components aggregate on the surface of the micro-polymerized hydrocolloid particles, thereby forming a gel-like blood clot that physically seals the bleeding point of the blood vessel. The local concentrations of platelets, coagulation factors, and fibrin increase significantly, subsequently triggering the endogenous coagulation mechanism (Burks and Spotnitz, 2014). At the same time, starch can be degraded by α-amylase into glucose, and its structure contains a large number of hydroxyl groups, which has excellent hydrophilicity.

However, MPH is commonly used in various surgical procedures such as general surgery and cardiothoracic surgery (Zhao et al., 2024; Wang et al., 2024; Bloom et al., 2021), but there are relatively few studies on its application in neurosurgery. In this work, an absorbable hemostatic powder was newly prepared by extracting from potato starch and followed by a series of chemical reactions such as cross-linking. The aim of this study was to investigate the effectiveness and biological safety of this absorbable hemostatic powder in stopping bleeding during neurosurgical operations for traumatic injuries to the brain both inside and outside the skull.

## 2 Materials and methods

### 2.1 Animals

A total of 18 conventional-grade Bama minipigs (9 males and 9 females), aged 3–4 months, weighing 20–30 kg, and in good health with normal physiological and biochemical parameters, were purchased from Guangdong Mingzhu Biotechnology Co., Ltd. (Production License: [SCXK(Yue) 2022-0061]; Quality Certificate No.: NO. 44828300000085). All animal breeding and experimental operation procedures have been approved by the Laboratory Animal Management and Use Committee of Hangzhou Lifutai Biotechnology Co., Ltd. (IACUC approval number: 20220716-01), and comply with the 3R principles of animal welfare.

### 2.2 Reagents

Potato starch (Sinopharm Chemical Reagent Co., Ltd.); sodium hydroxide, anhydrous ethanol (AR, Guangdong Guanghua Sci-tech Co., Ltd.); Span 80 (CP, Guangdong Guanghua Sci-tech Co., Ltd.); light liquid paraffin (Jilin Jihua Jiangcheng Oils and Chemicals Co., Ltd.); epichlorohydrin (Tianjin Kemiou Chemical Reagent Co., Ltd.); ethyl acetate (AR, Huzhou Shuanglin Chemical Technology Co., Ltd.); α-amylase, glucoamylase (Aladdin). Mouse fibroblast cell line L-929 (source of cells: Cell Bank of the Chinese Academy of Sciences); 1× MEM medium (Gibco); MTT reagent (Promega); 0.9% sodium chloride injection (Cisen Pharmaceutical Co., Ltd.); cottonseed oil (Acros Organics); propofol injection (Guangdong Jiabo Pharmaceutical Co Ltd.); isoflurane (RWD Life science Co., Ltd.). Detection kit (Siemens Healthineers): plasma prothrombin time (PT), activated partial thromboplastin time (APTT), plasma thrombin time (TT), fibrinogen (FBG). Masson staining reagents: Ponceau S (Sigma), acid fuchsin (Macklin), phosphomolybdic acid (Macklin), Aniline Blue (Shanghai Yuanye Bio-Technology Co., Ltd.). Medical gauze dressing (Jiaozuo Lianmeng Medical Materials Co., Ltd.). HaemoCer™ Hemostatic Powder (BioCer Entwicklungs-GmbH).

### 2.3 Instruments

NICOLET IS5 Fourier Infrared Spectrometer (ThermoFisher); ZEISS Sigma 300 Field Emission Scanning Electron Microscope (Germany); Brurker D8 Advance X-ray Diffractometer (Germany); AL204 Electronic Balance (Mettler-Toledo); DHG 9245A Electric Thermostatic Blast Oven (Shanghai Yiheng Technology Instrument Co.,Ltd.); TS-110DW Water Bath Thermostatic Shaker (Changzhou Jintan Jingda Instrument Co., Ltd.); H-1650 Benchtop High-Speed Centrifuge (Shanghai Lixinjian Centrifuge Co., Ltd.); TC300KA Electronic Balance (Changshu G&G Measurement Plant); FA2004 Electronic Balance (Shanghai SUNNY HENGPING Scientific Instrument Co., Ltd.); R620-S1-IECS General-Purpose Animal Anesthesia Machine (RWD Life Science Co., Ltd.); Automated Coagulation Analyzer (Sysmex Corporation, Japan); GEMINI AS Stainer (Thermo Fisher Scientific, USA); 120P Digital Slide Scanner (Shanghai Taohan Medical Technology Co., Ltd.)

### 2.4 Preparation and characterization of absorbable hemostatic powder

#### 2.4.1 Preparation of absorbable hemostatic powder

##### 2.4.1.1 Gelatinization and alkalization

Potato starch was dissolved in 45 °C water for injection to prepare a 40% starch aqueous solution, then added the solution to the vacuum stirring tank. The spiral shaft frictionally and hydraulically pressed the starch, generating heat and undergoing gelatinization, resulting in a white, thin, and runny paste-like substance. The gelatinization temperature ranged from 60 °C to 70 °C. Subsequently, the starch paste was alkalized by the dropwise addition of a 1 mol/L sodium hydroxide solution under continuous stirring.

##### 2.4.1.2 Emulsification

The light liquid paraffin and Span 80 were mixed in a vacuum emulsification mixer at a mass ratio (w/w) of 35:1 and stirred for 15 min the alkalized starch paste was slowly added and emulsified and stirred for 20 min. The temperature was set at 55 °C–60 °C and maintained with constant-speed stirring.

##### 2.4.1.3 Crosslinking reaction

When the temperature reached 55 °C, a certain amount of epichlorohydrin was added, and the mixture was stirred continuously. The reaction was carried out for 2 h while maintaining the temperature at 55 °C–60 °C. After the reaction was completed, the reaction solution was left at room temperature for 20 h to allow phase separation into two distinct layers.

##### 2.4.1.4 Drying and sieving

**FIGURE 1:**
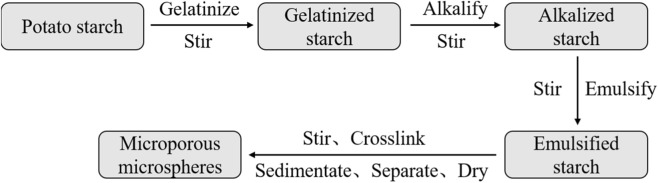
Process flowchart for preparing absorbable hemostatic powder

The resulting mixture was allowed to stand for phase separation, after which the upper clear layer was discarded and the lower milky liquid layer was collected. To the collected liquid, ethyl acetate was added as an extraction solvent at a volume ratio (v/v) of 1:5 under thorough stirring. After allowing the mixture to stand for another phase separation, the lower milky liquid layer was again collected. Subsequently, anhydrous ethanol was added for washing at 1:3 (v/v), with continuous stirring. Then, the mixture was subjected to vacuum filtration to remove moisture, yielding white particulate solid material. The obtained solid material was vacuum-dried at 65 °C–75 °C, sieved through a 150–360 mesh microporous sieve, and finally aseptically packaged and sterilized to obtain the absorbable hemostatic powder. The process flow is shown in Figure 1.

#### 2.4.2 Infrared spectroscopy analysis

An appropriate amount of the dried absorbable hemostatic powder was placed in an agate mortar. Then, added dried potassium bromide, and the mixture was thoroughly mixed and ground. After grinding evenly, a portion of the mixture was transferred into a tablet press mold and pressed into tablets. Finally, the pressed samples were carefully placed into the sample holder of the infrared spectrometer for testing. The absorbance of the samples was measured in the wavelength range of 4000 cm^-1^ to 400 cm^-1^.

#### 2.4.3 Crystal structure analysis

The crystalline structure of the dried absorbable hemostatic powder was analyzed by X-ray diffraction (XRD), with the following parameters set: the test material was copper (Cu), the diffraction angle range was 2*θ* = 5°–90°, and the scanning speed was 5°/min. The crystalline structure of the absorbable hemostatic powder was observed.

#### 2.4.4 Measurement of porosity and particle size distribution

The pore size distribution and porosity of the dried absorbable hemostatic powder were determined using a fully automatic mercury pressure apparatus. The apparatus operated within a pressure range of 0.1–61,000 psia and measured pore sizes ranging from 0.005 to 340 μm. The pore size distribution of the absorbable hemostatic powder was statistically analyzed, and its porosity was subsequently calculated.

#### 2.4.5 Microscopic morphology characterization

An appropriate amount of the dried absorbable hemostatic powder was evenly sprinkled onto a glass slide pre-coated with conductive adhesive. After the conductive glue fully adhered to the sample, an air blower was used to gently blow off the excess powder on the surface. Subsequently, gold spraying treatment was performed, and the sample was placed into the sample chamber of the scanning electron microscope for observation. The microscopic structure and morphology of the absorbable hemostatic powder were observed under different magnification levels.

#### 2.4.6 Water absorption rate and water absorption speed

##### 2.4.6.1 Water absorption rate

The centrifuge tube was weighed (designated as *M*_0_), and an appropriate amount of hemostatic powder (designated as *M*_1_) was introduced into the tube. An adequate volume of purified water was added to achieve complete saturation of the sample, followed by thorough shaking to ensure homogeneous hydration. The mixture was allowed to stand for 1 h, after which the supernatant was carefully removed with a dropper. The centrifuge tube containing the hydrated sample was weighed (designated as *M*_2_). The water absorption rate was calculated according to Formula 1.

$$\text{Water absorption rate}\%=\frac{M_{2}-M_{0}-M_{1}}{M_{1}}\times 100\%$$ (1)

##### 2.4.6.2 Water absorption speed

The water absorption speed of the hemostatic powder was determined by a contact angle measuring instrument. Water was dropped onto the surface of the hemostatic powder, and the time for the water droplet to disappear was observed.

#### 2.4.7 *In vitro* degradation performance

The *in vitro* degradation rate of hemostatic powder was characterized by combining the dialysis method with the weightlessness method. An appropriate amount of hemostatic powder was weighed (*W*_0_) into a stoppered conical flask, 0.5% α-amylase and 0.1% glucoamylase-PBS solution for dilution were added. The mixture was shaken well and then incubated in a water bath constant temperature shaking box (37 °C, 100 rpm) for enzymatic hydrolysis for 0.2 h–48 h respectively. The sample solutions from different time periods were transferred into 2000 Da dialysis bags. A large capacity beaker (filled with purified water) was used, and the dialysis bag containing the sample was placed in the beaker. The purified water in the beaker was replaced at intervals of 1 h, 2 h, 2 h, and 2 h, and left it overnight. The next day, the residue in the dialysis bag was taken and put it into the freeze-drying machine for −60 °C freeze-drying for 2–3 days. It was then taken out and weighed (*W*_1_). The percentage of weight loss (*W*) at each point was calculated according to Formula 2.

$$W\%=\frac{W_{0}-W_{1}}{W_{0}}\times 100\%$$ (2)

### 2.5 Biocompatibility evaluation

#### 2.5.1 Cytotoxicity test

According to GB/T 16886.5–2017“Biological evaluation of medical devices-Part 5: Tests for *in vitro* cytotoxicity” (GB/T 16886.5-2017, 2017), the cytotoxicity test was conducted on the absorbable hemostatic powder. The extraction medium (1×MEM complete medium containing 10% fetal calf serum, 100 IU/mL penicillin, 100 μg/mL streptomycin, and 4 mmol/L L-glutamine) was added at a ratio of 0.1 g/mL relative to the dry weight of the sample. The mixture was then placed in a 100 rpm shaking incubator for 72 h at 37 °C to prepare the extraction solution. The vigorously growing mouse fibroblast cells (L929) were digested and prepared into a cell suspension of 1 × 10^5^ cells/mL. A 96-well plate was prepared, and 100 μL of cell suspension was added to each well. The plate was then cultured at 37 °C in a 5% CO_2_ environment for 24 h. After discarding the original culture medium, 100 μL of each concentration sample (100%, 75%, 50% and 25%, v/v) of the extract, the negative control (sterilized 316 stainless steel material extract), and the positive control (5 g/L phenol 1×MEM complete medium solution) were added. A group with fresh culture medium alone was as the blank control group. At least 6 replicate wells were set up for each group. The cells were cultured in a 5% CO_2_ environment at 37 °C for 24 h. After 24 h of culture, the cell morphology was observed. All the liquid was removed from the 96-well plate, and 50 μL of MTT solution was added to each well. The plate was incubated at 37 °C for 2 h. Subsequently, the MTT solution was removed, and 100 μL isopropanol was added to each well. The microplate was gently shaken, and the absorbance at 570 nm was measured using an enzyme detector with a reference wavelength of 650 nm. The cell survival rate was calculated according to Formula 3.

$$\text{Cell survival rate}\left(\%\right)=\frac{{OD}_{570e}}{{OD}_{570b}}\times 100\%$$ (3)

In the formula: OD_570e_ - the average optical density of the 100% extracted sample; OD_570b_ - the average optical density of the blank.

#### 2.5.2 Acute systemic toxicity test

According to GB/T 16886.11–2021“Biological evaluation of medical devices - Part 11: Test for systemic toxicity” (GB/T 16886.11-2021, 2022), the acute systemic toxicity test was conducted on the absorbable hemostatic powder. The extract was prepared by adding 0.9% sodium chloride injection (10 mL) at a ratio of 0.1 g/mL of sample dry weight. The mixture was placed in a 37 °C constant-temperature shaker and subjected to horizontal oscillation at 100 rpm for 72 h to prepare the 0.9% sodium chloride injection extract. The cottonseed oil extract was prepared using the same ratio and extraction conditions. During the experiment, healthy mice were divided into 4 groups, with 5 mice in each group. 2 groups were injected with the extract solution of 0.9% sodium chloride (SC) and the control solution respectively into the abdominal cavity, with an injection volume of 50 mL/kg. The control solution was the extraction medium (without the test sample); the other two groups were injected with the cottonseed oil extract solution (CSO) and the control solution respectively into the abdominal cavity, with an injection volume of 50 mL/kg. The control solution was the extraction medium (without the test sample). Before injecting the sample extract, the body weight of each animal was measured and recorded. Immediately after the injection, the animals’ biological reactions were observed. At 4 h, 24 h, 48 h and 72 h post-injection, the general condition, toxic manifestations and the number of dead animals were observed and recorded for both the experimental group and the control group. Additionally, the body weight of the animals was measured and recorded at 24 h, 48 h and 72 h.

### 2.6 Animal experiment evaluation

#### 2.6.1 Establishment of animal model

Based on the surgical method for partial resection of the frontal lobe in Bama miniature pigs, a model of traumatic intracranial hemorrhage in small pigs was established. Before the operation, intravenous injection of 2 mg/kg propofol was administered via the ear marginal vein to induce general anesthesia. Following successful intubation and connection to the gas anesthesia ventilator, anesthesia was maintained with 2%–3% isoflurane. They were placed in a prone position for restraint. The hair in the surgical area of the head was shaved, and the skin was disinfected. The right frontal U-shaped flap was separated layer by layer. A bone window of approximately 4 cm × 3 cm was cut in the dura mater, and about 3 cm × 2 cm × 1 cm of right frontal lobe tissue was removed.

#### 2.6.2 *In vivo* hemostasis effect

##### 2.6.2.1 Experimental grouping

18 Bama miniature pigs, with equal numbers of males and females, were randomly divided into three groups: the model control group (medical gauze dressing), the experimental group (absorbable hemostatic powder), and the marketed control group (HaemoCer™ hemostatic powder), with 6 pigs in each group (3 males and 3 females per group).

##### 2.6.2.2 Observation indicators

The absorbable hemostatic powder and HaemoCer™ hemostatic powder were respectively sprayed onto the craniocerebral wounds of the experimental group and the marketed control group. The observation was conducted for 15 s, if there was still relatively rapid blood oozing, additional hemostatic powder was applied to the oozing area until no obvious oozing was observed. Then, a medical gauze dressing was used for gentle pressure, and the medical gauze dressing was gently removed every 5 s to assess the hemostasis situation. After the bleeding stops, the medical gauze dressing was removed, and the time of hemostasis and the amount of bleeding were recorded respectively.

1. Hemostatic time: Record the total time required to achieve the bleeding cessation effect, in seconds (s).
2. Bleeding volume: Use sterile gauze to collect all the bleeding that occurs during the hemostasis process, and measure it in grams (g).
3. Dosage: Weigh the dosage of hemostatic powder required for the experimental group and the marketed control group respectively to achieve the hemostatic effect, and record the usage in grams (g) by weight.

#### 2.6.3 Safety research

##### 2.6.3.1 Experimental grouping

1. Sham operation group: Only scalp incision was performed without craniotomy, 6 animals, with an equal number of males and females.
2. Experimental Group (Absorbable Hemostatic Powder): A model of intracranial and extracranial traumatic hemorrhage was established by partial resection of the frontal cortex. The 12 animals were derived from model control group (6 animals) and 6 experimental group (6 animals) in the hemostatic efficacy study, with an equal number of males and females.
3. Marketed control group (HaemoCer™ hemostatic powder): A model of intracranial and extracranial traumatic hemorrhage was established by partial resection of the frontal cortex. 6 animals were from the marketed control group animals (6 animals) used in the hemostatic efficacy study, with an equal number of males and females.

##### 2.6.3.2 Observation indicators

After the bleeding has stopped, the hemostatic powder at the wound site was moistened with a wet gauze, then the skin on the head was sutured, and the animal was returned to the cage for observation of its survival status. For the next 3 days after the operation, 1.6 million IU of penicillin sodium was administered by intramuscular injection and 4 mg/kg of ibuprofen tablets were taken orally to provide anti-inflammatory and analgesic treatment after the operation.

In the experimental group, 3 animals were treated respectively at 7 days and 14 days after the operation. The degradation and absorption of the wound surface and materials were visually observed, and tissue were collected samples from the wound surface for histopathological examination. At 28 days after the operation, 2 animals from each of the sham operation group, the experimental group and the marketed control group were subjected to the respective treatments. The remaining 4 animals in each group were further observed for an additional 42 days.

1. General physical condition observation: After the operation, the general condition of the animals such as their mental state, behavior, activity, and diet, as well as the healing status of the wounds, was observed. Simultaneously, the body weight and body temperature were recorded on the 1 d, 3 d, 7 d, 14 d, 21 d, 28 d, and 42 d after the operation.
2. Blood coagulation indicators: At the preoperative and postoperative time points of 1 day, 3 days, 7 days, 14 days, 21 days, 28 days, and 42 days, after fasting for 12 h, the anterior vena cava blood of the animals was collected in an empty state and mixed with 2 mL of 3.8% citrate sodium for anticoagulation (1:9). The changes in fibrinogen time (FBG-T), fibrinogen content (FBG), plasma thrombin time (TT), plasma prothrombin time (PT), prothrombin activity (%PT), PT international normalized ratio (INR), and activated partial thromboplastin time (APTT) were determined on an automatic coagulation analyzer to evaluate the tissue compatibility and safety of the reaction materials in the body.
3. General observation and pathological tissue examination of the wound: The experimental group treated 3 animals at 7 days and 14 days after the operation respectively. The experimental group, the marketed control group and the sham operation group each handled 2 animals and 4 animals respectively at 28 days and 42 days after the operation. The euthanasia of the miniature pigs was performed using excessive anesthesia combined with auxiliary bloodletting. The propofol (15 mg/kg) was injected via the marginal ear vein to ensure complete anesthesia of the miniature pigs, as evidenced by muscle relaxation and absence of corneal reflex. Subsequently, the pigs were placed in a lateral position and secured on the dissection table. The skin over the femoral triangle was incised, and the femoral artery and vein were severed. The incision site was rinsed with warm water to prevent blood clotting until cessation of vital signs was confirmed. Following euthanasia, a general observation was made of the healing condition of the brain tissue wound. Whether there were any residual materials, whether there was any secondary bleeding or blood clots, etc., are checked. At the same time, brain tissues from the area where the hemostatic powder was applied were also taken. These tissues were fixed with 4% paraformaldehyde, embedded, and sectioned into paraffin blocks. They were then subjected to histopathological staining with hematoxylin-eosin (H&E) to observe the pathological changes of the brain tissues in the area where the hemostatic powder was applied.

#### 2.6.4 Statistical analysis

All the measured data were expressed as mean ± standard deviation (x̄ ± s). Statistical analysis was performed using IBM SPSS 22.0 software. One Way ANOVA was used for the analysis. If the variances were homogeneous, the Tukey method was used for pairwise comparisons of the means of multiple groups. If the variances were not homogeneous, the Dunnett’s T3 method was used instead. The independent sample T-test was used to compare the experimental group with the corresponding control group. *P* < 0.05 indicated a statistically significant difference.

## 3 Results

### 3.1 Characterization of absorbable hemostatic powder

#### 3.1.1 Infrared spectroscopy analysis

**FIGURE 2:**
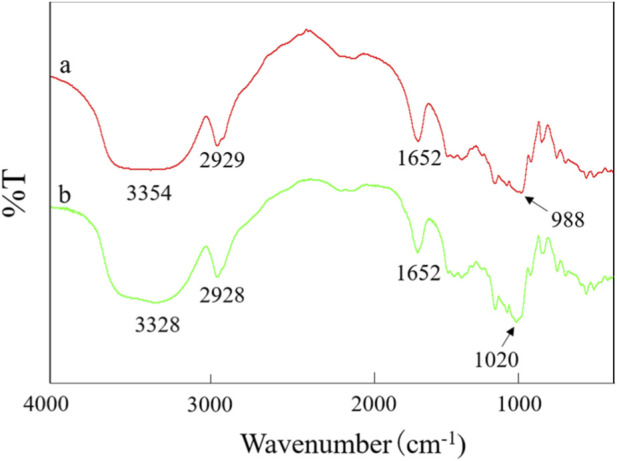
Infrared spectrum of **(a)** potato starch and **(b)** absorbable hemostatic powder.

The infrared spectrum is shown in Figure 2. For potato starch (a) and absorbable hemostatic powder (b), there were extremely strong and broad absorption peaks at 3,354 cm^-1^ and 3,328 cm^-1^ respectively, mainly representing the characteristic absorption peaks of hydroxyl groups (-OH) in starch molecules (Marinopoulou et al., 2025; Wu et al., 2020); characteristic absorption peaks were also observed around 2,930 cm^-1^ and 1,652 cm^-1^. As can be seen from Figure 2, the infrared spectra of the absorbable hemostatic powder and the potato starch raw material were similar, indicating that the main functional groups contained in both were the same. Furthermore, from Figure 2, it was observed that potato starch (a) exhibited an absorption peak at 988 cm^-1^, indicating the presence of a crystalline starch structure (Sevenou et al., 2002)^.^ In contrast, the absorption peak of the cross-linked absorbable hemostatic powder (b) disappeared at this wavelength, while a new absorption peak appeared at 1,020 cm^-1^, which is the amorphous region of starch and corresponds to the random coil structure of the starch macromolecules (Marinopoulou et al., 2025). This observation suggests that the amorphous structure of the cross-linked absorbable hemostatic powder has increased.

#### 3.1.2 Crystal structure analysis

**FIGURE 3:**
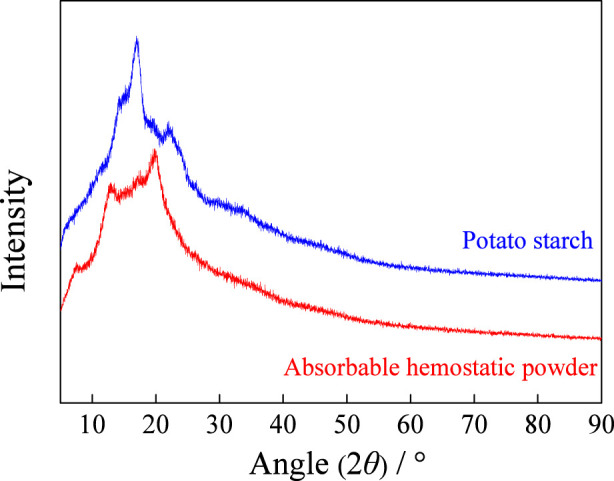
XRD of potato starch and absorbable hemostatic powder.

X-ray diffraction (XRD) analysis reveals that the structure of starch granules is mainly composed of two nearly equal parts, namely, the disordered amorphous region and the ordered crystalline region. Figure 3 shows the X-ray diffraction patterns of potato starch and absorbable hemostatic powder. As can be seen from Figure 3, a significant diffraction peak of potato starch appeared at 2*θ* of 17°, and a clear diffraction peak was also observed near 2*θ* = 22°, indicating that this starch exhibited the typical B-type X-ray diffraction pattern characteristic of root and tuber starch (significant diffraction peaks at 5°, 17°, 22°, and 24°) (Tang et al., 2006). However, it is worth noting that the intensity distribution of the diffraction peaks of type B starch may vary slightly depending on the plant species or growth conditions. The 17° peak of potato starch was significantly stronger than the 5° peak, suggesting that its crystal arrangement may be more in line with the partially ordered characteristics of type A starch (15°, 17°, 18°, 23°) (Tang et al., 2006)^,^ but it is still classified as type B overall.

When potato starch is heated in excessive water to the gelatinization temperature, the starch particles will expand and the crystal structure will gradually be disrupted. Subsequently, the starch molecules cross-link and aggregate to form microspheres, and the crystalline structure changes. In the X-ray diffraction analysis, the absorbable hemostatic powder showed strong diffraction peaks near 2*θ* = 12.5° and 19.5°, along with a minor peak at 7.8°, presenting a V-shaped diffraction pattern (Tang et al., 2006). In contrast, the peak shape of potato starch was relatively sharp, and the area ratio of the diffraction peaks was higher than that of the absorbable hemostatic powder, indicating that the crystallinity of potato starch decreases after being processed into absorbable hemostatic powder. This also proves that the proportion of the amorphous region of the absorbable hemostatic powder increases, which is beneficial to the water absorption performance of the absorbable hemostatic powder.

#### 3.1.3 Measurement of porosity and particle size distribution

**FIGURE 4:**
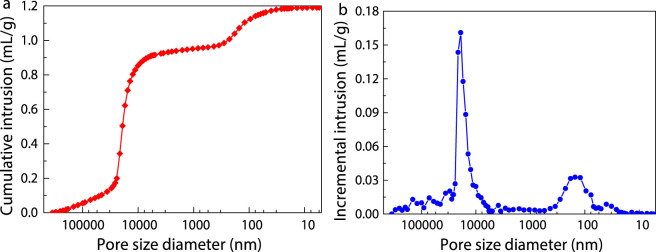
**(a)** Cumulative intrusion and **(b)** incremental intrusion of absorbable hemostatic powder as a function of pore size.

Porosity and pore size distribution as key performance parameters of porous hemostatic materials, exerting a decisive influence on hemostasis speed and the ultimate hemostatic effect. Figure 4a illustrates the dynamic relationship between the cumulative intrusion volume of absorbable hemostatic powder and pore size. The cumulative intrusion volume metric essentially provides a quantitative insight into the capacity of the material’s internal pore structure to hold intruding substances. From the curve characteristics in Figure 4a, it was evident that within the range of smaller pore sizes, the cumulative intrusion volume exhibited a relatively gradual trend. Nevertheless, once the pore size increased to a specific range, the cumulative intrusion volume demonstrated a sharply changing pattern. This significant change suggests the presence of numerous large-pore structures within the absorbable hemostatic powder, which play a pivotal role during the filling process by intruding substances. Utilizing the calculation approach based on the ratio of the total intrusion volume to the total material volume, it was determined that the absorbable hemostatic powder has a high porosity of 61.77%, indicating that the material has a highly developed and complex pore structure system.

Figure 4b depicts the distribution of the invasion intrusion within pores of the absorbable hemostatic powder across varying pore sizes. Figure 4b unequivocally demonstrated that the pore size distribution of the absorbable hemostatic powder was primarily bimodal, with peaks corresponding to pore diameters of 19 μm and 151 nm. The peak corresponding to a pore diameter of 19 μm was the most prominent, indicating that this size of pore constituted a large proportion of the overall pore structure. The research results showed that the high porosity of the absorbable hemostatic powder, combined with its multi-peak pore size distribution, significantly increased the contact area between the material and blood. The larger contact area facilitates rapid hemostasis and enhances the hemostatic effect.

#### 3.1.4 Scanning electron microscopy observation

**FIGURE 5:**
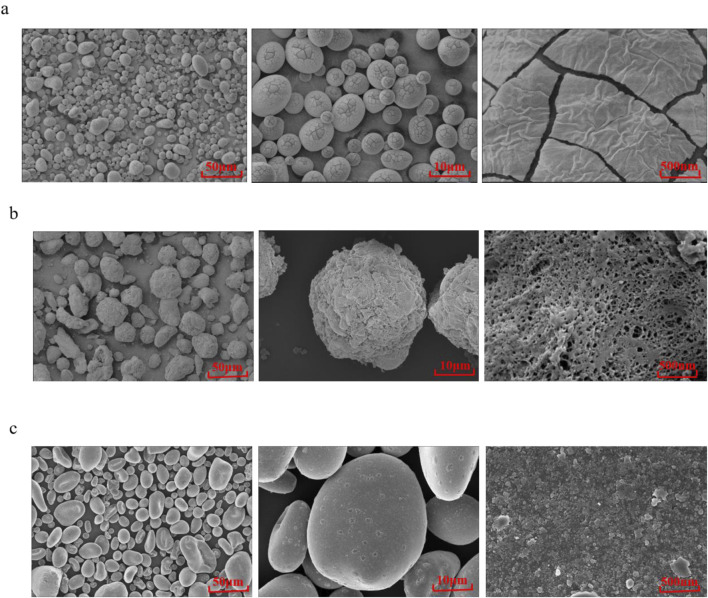
SEM of **(a)** potato starch, **(b)** absorbable hemostatic powder and **(c)** HaemoCer™ hemostatic powder.

Figure 5 presents scanning electron microscope (SEM) images of potato starch raw material (a), absorbable hemostatic powder (b) and HaemoCer™ hemostatic powder (c). The surface of the potato starch was relatively smooth, dense, and exhibited uniform cracks, but no pore structure was observed (Figure 5a). In contrast, the surface of the cross-linked absorbable hemostatic powder was rough, uneven, and featured pore structures with varying dimensions, resulting in a three-dimensional network architecture within the material (Figure 5b). The multi-level interconnected pore of the cross-linked hemostatic powder can significantly enhance the material’s specific surface area, enabling rapid absorption of water from blood and concentrate of solid components. This property contributes to improved hemostatic performance. However, the surface of HaemoCer™ hemostatic powder appeared granular and no obvious pore structure was observed (Figure 5c).

#### 3.1.5 Water absorption rate and water absorption speed

**FIGURE 6:**
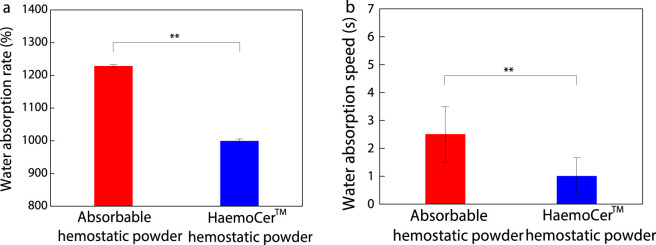
**(a)**Water absorption rate and **(b)**water absorption speed of absorbable hemostatic powder and HaemoCer™ hemostatic powder (Note: Compared with HaemoCer™ hemostatic powder, ^**^ *P* < 0.01.).

The water absorption property of hemostatic powder is a crucial indicator for its effectiveness use as a hemostatic material. Upon water absorption, the material concentrates platelets and coagulation factors in blood, thereby accelerating the coagulation process (Hickman et al., 2018). Figure 6 presents the water absorption rate and speed of the absorbable hemostatic powder and HaemoCer™ hemostatic powder. As shown in Figure 6a, the absorbable hemostatic powder exhibited a water absorption capacity exceeding 1,200%, whereas the HaemoCer™ hemostatic powder reached approximately 1,000%. This indicates that the absorbable hemostatic powder has a significantly higher water absorption capacity than that of HaemoCer™ hemostatic powder (*P* < 0.01). In terms of water absorption speed (Figure 6b), the absorbable hemostatic powder demonstrated a slower absorption rate compared to the HaemoCer™ hemostatic powder (*P* < 0.01).

#### 3.1.6 *In vitro* degradation performance

**FIGURE 7:**
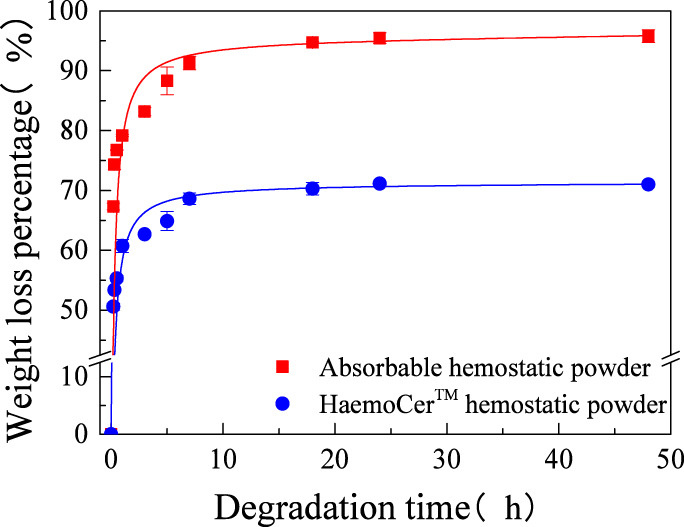
*In vitro* degradation curve of absorbable hemostatic powder and HaemoCer™ hemostatic powder.

An *in vitro* degradation test was conducted on the hemostatic powder under simulated physiological conditions. The results are shown in Figure 7. The weight loss percentage of the absorbable hemostatic powder increased rapidly within the first 7 h, after which the degradation rate slows down. The degradation rate of the absorbable hemostatic powder at 7 h was 91.26%, and it exceeded 95% after 48 h, suggesting that the absorbable hemostatic powder is essentially completely degraded within this timeframe, with almost no residual material remaining after the *in vitro* degradation process. The degradation trend of HaemoCer™ hemostatic powder exhibited similar to that of absorbable hemostatic powder. However, at 48 h, the degradation rate of HaemoCer™ hemostatic powder remained below 70%, and under identical degradation conditions, its weight loss percentage consistently remained lower than that of absorbable hemostatic powder. This difference may be attributed to the distinct cross-linking structures of the two materials. The HaemoCer™ hemostatic powder contains substituents (carboxymethyl groups), which modify the spatial configuration of the polymer chain segments, thereby conferring enhanced resistance to enzymatic degradation (Yoshida and Kishikawa, 2010). Consequently, its degradation behavior differs from that of the absorbable hemostatic powders.

Through data fitting, it was found that the *in vitro* degradation process of both materials conformed to the Nelder relevant kinetic model, with Formula 4 shown as follows.

$$y=\frac{\left(x+a\right)}{\left(b_{0}+b_{1}\times \left(x+a\right)+b_{2}\times {\left(x+a\right)}^{2}\right)}$$ (4)

By using the experimental data to solve the parameters in the kinetic model, it was found that the *R* ^2^ of the model is ≥0.98 at different time points. This indicates that the established model can effectively simulate the changing pattern of the degradation rate of the samples over time, gradually increasing as the degradation time progresses.

### 3.2 Biocompatibility evaluation

#### 3.2.1 Cytotoxicity test

**FIGURE 8:**
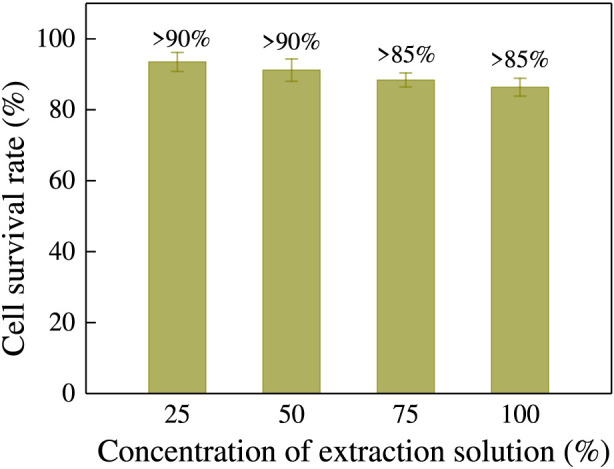
Cytotoxicity of absorbable hemostatic powder against L929 cells incubation.

The cytotoxicity of different concentrations of absorbable hemostatic powder on mouse fibroblast L929 was evaluated using the MTT assay. After 24 h of incubation, the cell layer in the positive control group was almost completely destroyed, with a cell survival rate of only 11.0%, whereas the negative control group exhibited a cell survival rate of 95.7%. Figure 8 shows the toxicity test results of the extract solutions of absorbable hemostatic powder at varying concentrations. The experimental results indicate that when the concentration of the extract solution reached 100% (v/v), the cell survival rate exceeded 85%; at concentrations below 50%, the cell survival rate remained above 90%. According to the biological evaluation standards for medical devices (GB/T 16886.5-2017, 2017), if the survival rate drops to less than 70% of the blank control in the MTT cytotoxicity test, it indicates potential cytotoxicity. Therefore, the absorbable hemostatic powder demonstrates no potential cytotoxicity and exhibits excellent biocompatibility.

#### 3.2.2 Acute systemic toxicity test

Table 1 presents the injection protocol for both groups: the 0.9% sodium chloride injection extract solution (SC) and the control solution are administered into the abdominal cavity at a dose of 50 mL/kg. The control solution consisted solely of the extraction medium (without the test sample). Table 2 details the injection procedure for the cottonseed oil extract (CSO) group, where the test solution is injected into the abdominal cavity, while the control group receives the control solution (the extraction medium without the test sample), also at 50 mL/kg. Following the injection, the biological responses of the animals were monitored. Based on the observed acute systemic toxic reactions, it was found that the body weight of the mice naturally increased within 72 h, with a growth trend consistent with as that of the control group. During 72-h observation period for toxic reactions, no obvious adverse effects were observed. The test results indicate that the absorbable hemostatic powder exhibits no acute systemic toxic reactions.

**TABLE 1:** Observation results of acute systemic toxicity (SC).

Drug-delivery way	No.	Sex	Weight/g				Observation of toxic reactions in animals after injection				
			Initial	After 24 h	After 48 h	After 72 h	Real time	After 4 h	After 24 h	After 48 h	After 72 h
Abdominal cavity (control group)	1	Male	18	21	21	22	—	—	—	—	—
	2	Male	19	21	22	23	—	—	—	—	—
	3	Male	19	22	23	24	—	—	—	—	—
	4	Male	20	22	24	25	—	—	—	—	—
	5	Male	21	22	24	25	—	—	—	—	—
Abdominal cavity (experimental group)	1	Male	18	19	20	22	—	—	—	—	—
	2	Male	19	19	20	23	—	—	—	—	—
	3	Male	19	20	22	24	—	—	—	—	—
	4	Male	20	22	23	25	—	—	—	—	—
	5	Male	21	22	24	26	—	—	—	—	—

“—“ Indicates normal condition and no toxic reaction was observed.

**TABLE 2:** Observation results of acute systemic toxicity (CSO).

Drug-delivery way	No.	Sex	Weight/g				Observation of toxic reactions in animals after injection				
			Initial	After 24 h	After 48 h	After 72 h	Real time	After 4 h	After 24 h	After 48 h	After 72 h
Abdominal cavity (control group)	1	Male	18	19	20	20	—	—	—	—	—
	2	Male	19	19	22	24	—	—	—	—	—
	3	Male	20	22	23	24	—	—	—	—	—
	4	Male	21	22	23	25	—	—	—	—	—
	5	Male	22	23	24	26	—	—	—	—	—
Abdominal cavity (experimental group)	1	Male	18	20	22	24	—	—	—	—	—
	2	Male	19	20	22	24	—	—	—	—	—
	3	Male	19	20	23	25	—	—	—	—	—
	4	Male	20	21	23	25	—	—	—	—	—
	5	Male	21	23	24	27	—	—	—	—	—

“—“ Indicates normal condition and no toxic reaction was observed.

### 3.3 Animal experiment evaluation

#### 3.3.1 Establishment of animal model

**FIGURE 9:**
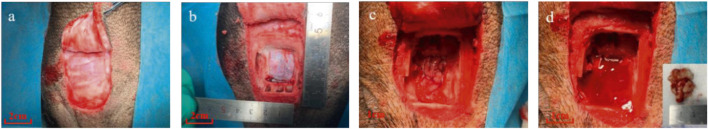
Establishment of intracerebral and intracerebral hemorrhage model of miniature pigs **(a)** right frontal U-shaped flap was separated after the surgical area was disinfected, **(b)** bone window of about 4 cm × 3 cm was formed, **(c)** cut open the meninges and stop the bleeding carefully, **(d)** remove the tissue in the right frontal lobe and visible bleeding was observed with the naked eye, the illustration shows the resected right frontal tissue.

The Bama miniature pig serves as an ideal humanoid animal model due to its similarity in blood volume to humans. To evaluate the hemostatic efficacy and safety of the absorbable hemostatic powder, a model combining external and internal brain injuries with active bleeding was established in miniature pigs. Prior to surgery, the animals were induced into general anesthesia positioned in a prone posture, and immobilized. The surgical area on the head was shaved and disinfected. A right frontal U-shaped flap was separated layer by layer. Subsequently, a bone window measuring approximately 4 cm × 3 cm was cut in the dura mater, and careful hemostasis was applied. Approximately 3 cm × 2 cm × 1 cm of tissue from the right frontal lobe was excised. Severe active bleeding points were controlled using electro-coagulated. The presence of persistent visible bleeding confirmed the successful establishment of the pig’s brain external and internal bleeding model (Figure 9).

#### 3.3.2 Hemostasis effect

**FIGURE 10:**
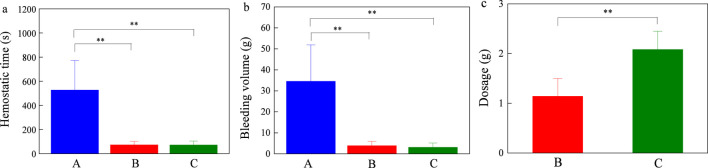
Hemostatic effect on intracranial and intracerebral injury bleeding model of miniature pigs **(a)** hemostatic time, **(b)** bleeding volume, **(c)** dosage of hemostatic powder (Note: Group A represents the model operation group; Group B represents the experimental group; Group C represents the already marketed control group. Compared with the model control group, ^*^ *P* < 0.01; comparison between the experimental group and the already marketed control group, ^*^ *P* < 0.05, ^**^ *P* < 0.01.).

The experimental results demonstrate that the absorbable hemostatic powder and HaemoCer™ hemostatic powder immediately adsorb the blood upon contact with the intracranial wound surface, forming an instant gel matrix that accelerate hemostasis. No secondary intracranial bleeding was observed in either group. As shown in Figure 10, compared with the model control group (medical gauze dressing), both the experimental group (absorbable hemostatic powder) and the already marketed control group (HaemoCer™ hemostatic powder) exhibited significantly reduced bleeding time and bleeding volume in small pigs with intracranial and extracranial traumatic bleeding (*P* < 0.01). Furthermore, there was no significant difference in bleeding time or bleeding volume between the experimental group and the marketed control group (*P* > 0.05). The average bleeding time of the experimental group was 73.50 ± 29.08 s, the amount of bleeding was 3.90 ± 2.09 g, while those of the marketed control group were 72.33 ± 32.14 s and 3.12 ± 2.03 g, respectively. This indicates that the absorbable hemostatic powder has a good hemostatic effect on capillary, artery and vein bleeding. Although there were no significant differences in the bleeding time and bleeding volume between the experimental group and the marketed control group, from the perspective of the dosage required to achieve the bleeding stoppage effect, the dosage used in the experimental group (absorbable hemostatic powder) was significantly less than that in the marketed control group (HaemoCer™ hemostatic powder) (*P* < 0.01). This was related to the different cross-linking structures of the two (Figure 4). The multi-microporous structure of the absorbable hemostatic powder increases the contact area of blood, enhancing the enrichment of local coagulation factors, and thereby ensures the therapeutic effect while reducing the material usage.

#### 3.3.3 Application security

##### 3.3.3.1 General physical condition observation

Through observations of general condition, body weight, and anal temperature, it was found that the mental state and activity of animals in the sham operation group were normal, with normal food and water intake, and they responded sensitively to external stimuli. In both the experimental group and the marketed control group, no limb paralysis or inability to fully extend, turn around or stand stably occurred in the small pigs after the operation. Food intake recovered within 2–3 days postoperatively. The animals’ mental state improved, and their activity increased by 7 days after surgery. No wound rupture, purulent infection, or other phenomena occurred during the postoperative period, and the prognosis was favorable. In addition, the body weights of the small pigs in each group decreased within 7 days postoperatively due to factors such as fasting and blood collection procedures. However, by 14 days, their weights had returned to pre-operation levels and gradually increased, with no significant differences among the groups (*P* > 0.05) (Table 3). Compared with the sham operation group, the rectal temperatures of the experimental group and the marketed control group were significantly higher on the third day after surgery (*P* < 0.05), but remained within the normal body temperature range. No significant differences were observed among the three groups at other time points (*P* > 0.05) (Table 3), indicating that the application of absorbable hemostatic powder does not have a significant impact on the animal’s physical condition.

**TABLE 3:** Changes of body weight (kg) and anal temperature (°C) of miniature pigs in each group (x̄ ± s).

Group	Weight/kg			Anal temperature/°C		
	Sham-operated group	Experimental group	Marketed control group	Sham-operated group	Experimental group	Marketed control group
Pre-operation	31.92 ± 2.57 (n = 6)	30.77 ± 3.87 (n = 12)	31.40 ± 3.23 (n = 6)	38.42 ± 0.32	38.40 ± 0.31	38.38 ± 0.25
Post-operation 1 day	31.48 ± 3.51 (n = 6)	29.46 ± 4.28 (n = 12)	30.34 ± 4.14 (n = 6)	38.42 ± 0.19	39.00 ± 0.64	38.95 ± 0.63
Post-operation 3 days	30.84 ± 3.26 (n = 6)	28.67 ± 4.12 (n = 12)	29.91 ± 3.45 (n = 6)	38.33 ± 0.16	39.09 ± 0.46**	39.17 ± 0.49**
Post-operation 7 days	32.40 ± 3.25 (n = 6)	30.30 ± 3.99 (n = 12)	30.71 ± 3.78 (n = 6)	39.28 ± 0.90	38.87 ± 0.68	39.47 ± 0.68
Post-operation 14 days	32.70 ± 3.32 (n = 6)	30.78 ± 3.36 (n = 9)	31.67 ± 3.49 (n = 6)	38.65 ± 0.79	38.74 ± 0.62	38.43 ± 0.31
Post-operation 21 days	33.15 ± 3.07 (n = 6)	32.23 ± 3.41 (n = 6)	32.63 ± 3.71 (n = 6)	38.47 ± 0.38	38.53 ± 0.38	38.50 ± 0.45
Post-operation 28 days	33.37 ± 3.08 (n = 6)	32.73 ± 3.29 (n = 6)	32.95 ± 3.72 (n = 6)	38.50 ± 0.33	38.90 ± 0.33	38.63 ± 0.39
Post-operation 42 days	33.48 ± 1.18 (n = 4)	34.38 ± 3.51 (n = 4)	34.03 ± 4.11 (n = 6)	38.55 ± 0.62	38.53 ± 0.40	38.68 ± 0.19

Compared with the sham operation group, *P < 0.05, **P < 0.01.

##### 3.3.3.2 Blood coagulation indicators

The fibrinolytic system serves as a crucial defense mechanism that dissolves fibrin deposits both inside and outside blood vessels, ensuring vascular patency and preventing thrombosis. Prothrombin time (PT), activated partial thromboplastin time (APTT), thrombin time (TT), and fibrinogen (FBG) assays are important indicators for evaluating pathological changes in the body’s hemostasis and coagulation systems, screening for coagulation-related disorders before surgery, and guiding clinical medication treatment. The research results (Table 4) indicated that compared with the sham operation group, the PT and INR in the experimental group were significantly increased at 1 day and 3 days after surgery (*P* < 0.05, *P* < 0.01), while the %PT was significantly decreased (*P* < 0.01). At 7 days, the FBG-T was significantly shortened (*P* < 0.05), and no significant effects were observed at other time points (*P* > 0.05). This might be related to the degree of bleeding in the animals of each group during the modeling process. However, after 7 days postoperatively, these abnormal blood coagulation indicators all returned to normal levels, indicating that the absorbable hemostatic powder does not exert obvious adverse effects on the blood coagulation system and exhibits good tissue compatibility and safety.

**TABLE 4:** Changes of hemagglutination indexes of miniature pigs in each group (x̄ ± s).

Index	Group	Pre-operation	Post-operation						
			1 day	3 days	7 days	14 days	21 days	28 days	42 days
PT (s)	1	12.27 ± 0.92	11.60 ± 0.95	11.22 ± 1.08	11.77 ± 0.80	11.83 ± 1.20	11.57 ± 1.30	11.12 ± 1.36	11.95 ± 0.83
	2	11.96 ± 0.75	14.11 ± 1.00^**^	12.39 ± 1.03*	12.23 ± 1.08	12.26 ± 1.02	12.57 ± 1.01	11.38 ± 0.91	11.13 ± 1.21
	3	11.52 ± 1.57	13.53 ± 1.15*	11.87 ± 0.19	11.97 ± 0.66	12.23 ± 0.27	12.30 ± 0.58	11.60 ± 1.46	12.23 ± 0.76
%PT (%)	1	77.58 ± 9.82	85.33 ± 12.42	90.57 ± 14.35	82.95 ± 9.47	82.97 ± 13.30	86.72 ± 16.92	92.72 ± 17.25	80.80 ± 8.75
	2	80.73 ± 8.67	61.49 ± 6.92^**^	76.60 ± 11.29*	78.38 ± 12.03	77.88 ± 10.20	74.65 ± 10.25	88.03 ± 13.09	92.10 ± 17.26
	3	88.45 ± 22.00	66.07 ± 8.54*	81.12 ± 2.12	80.43 ± 7.25	77.18 ± 2.84	76.75 ± 6.04	86.87 ± 19.28	77.73 ± 8.22
INR	1	1.07 ± 0.09	1.01 ± 0.09	0.97 ± 0.10	1.03 ± 0.07	1.03 ± 0.11	1.01 ± 0.12	0.97 ± 0.12	1.04 ± 0.08
	2	1.04 ± 0.07	1.24 ± 0.09**	1.08 ± 0.10*	1.07 ± 0.10	1.07 ± 0.09	1.10 ± 0.09	0.99 ± 0.08	0.97 ± 0.11
	3	1.00 ± 0.14	1.19 ± 0.11*	1.04 ± 0.02	1.04 ± 0.06	1.07 ± 0.02	1.07 ± 0.05	1.01 ± 0.13	1.07 ± 0.07
APTT (s)	1	23.02 ± 3.51	22.15 ± 2.89	20.22 ± 2.69	20.85 ± 1.47	20.00 ± 2.59	20.90 ± 2.73	19.88 ± 2.26	20.55 ± 1.58
	2	21.61 ± 3.13	23.18 ± 2.19	19.72 ± 2.83	19.13 ± 3.26	18.61 ± 2.11	21.63 ± 1.10	18.10 ± 2.56	17.13 ± 1.89
	3	20.30 ± 3.27	23.22 ± 2.58	19.52 ± 2.52	18.35 ± 2.57	19.08 ± 1.38	20.52 ± 2.83	19.82 ± 2.23	20.28 ± 1.60
TT (s)	1	19.32 ± 1.76	17.42 ± 2.24	18.22 ± 1.36	18.22 ± 0.97	17.88 ± 1.86	19.55 ± 1.11	20.25 ± 2.22	19.60 ± 0.80
	2	19.10 ± 1.27	18.28 ± 0.72	18.23 ± 1.85	18.18 ± 2.65	18.46 ± 1.28	19.60 ± 1.17	19.48 ± 3.78	18.58 ± 2.00
	3	17.02 ± 3.53	18.48 ± 0.81	17.98 ± 0.70	16.95 ± 1.62	18.20 ± 0.94	19.02 ± 0.70	19.63 ± 1.68	19.70 ± 1.13
PBG-T (s)	1	10.50 ± 4.24	6.77 ± 1.05	7.77 ± 0.78	9.88 ± 0.90	11.67 ± 2.62	13.92 ± 1.93	12.73 ± 2.38	9.70 ± 1.61
	2	11.88 ± 1.95	5.76 ± 0.97	6.67 ± 2.63	7.12 ± 2.05*	9.47 ± 2.61	11.55 ± 3.52	12.10 ± 2.37	9.53 ± 2.34
	3	11.45 ± 3.23	6.13 ± 0.96	5.70 ± 0.88	6.88 ± 1.62*	10.23 ± 2.38	12.98 ± 1.01	11.68 ± 2.70	10.53 ± 1.21
FBG (g/L)	1	1.95 ± 1.20	2.70 ± 0.47	2.26 ± 0.30	1.69 ± 0.18	1.47 ± 0.61	1.13 ± 0.20	1.28 ± 0.28	1.75 ± 0.31
	2	1.39 ± 0.32	3.42 ± 1.00	3.10 ± 1.28	2.72 ± 0.98	1.93 ± 0.66	1.55 ± 0.65	1.36 ± 0.32	1.85 ± 0.54
	3	1.55 ± 0.62	3.05 ± 0.57	3.36 ± 0.68*	2.80 ± 0.98	1.70 ± 0.49	1.21 ± 0.12	1.46 ± 0.49	1.57 ± 0.23

Group 1 represents the sham operation group; Group 2 represents the experimental group; Group 3 represents the marketed control group. Compared with the sham operation group, *P < 0.05, **P < 0.0.1.

##### 3.3.3.3 General observation

**FIGURE 11:**
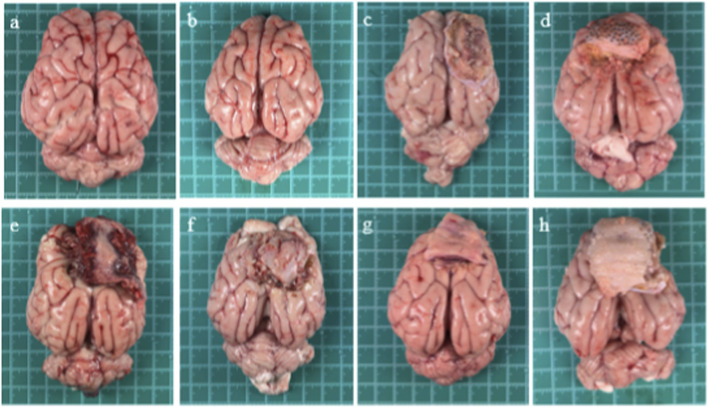
General observation of intracerebral wounds of miniature pigs. **(a)** 28 days and**(b)**42 days after operation in sham group. **(c)** 28 days and **(d)** 42 days after the operation in marketed control group. **(e)** 7 days, **(f)** 14 days, **(g)** 28 days and **(h)** 42 days after operation in experimental group.

A general observation was made on the treatment points at different times after the operation. The skin incisions of the animals in each group healed well, and no infection occurred. No cerebrospinal fluid or abscesses were observed in the brain. At 7 days after the operation, a thin fibrous membrane was observed on the surface of the wound in the experimental group. There were still many materials and blood forming red gel matrix components within the wound, and no original form was seen (Figure 11e). At 14 days after the operation, the wound in the experimental group was covered by fibrous connective tissue, and a small amount of gel matrix components were still present in the wound (Figure 11f). At 28 days after the operation, no abnormalities were observed in the brain tissue of the sham operation group (Figure 11a). In the experimental group and the marketed control group, granulation tissue had formed on the wound surface, and no materials were left visually (Figures 11c,g). At 42 days after the operation, no abnormalities were observed in the brain tissue of the sham operation group (Figure 11b). In the experimental group and the marketed control group, the wound surfaces had been filled, organized and repaired by granulation tissue, and no materials were left visually (Figures 11d,h).

##### 3.3.3.4 Observation of pathological tissues of the wound surface

**FIGURE 12:**
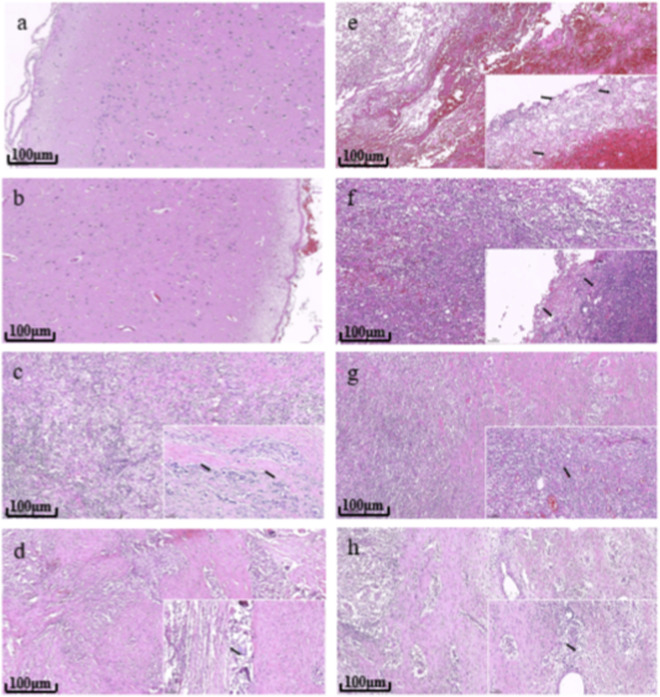
Histopathological observation of wound brain tissue (100×). **(a)** 28 days and **(b)**42 days after operation in sham group. **(c)** 28 days and **(d)** 42 days after the operation in marketed control group. **(e)** 7 days, **(f)** 14 days, **(g)** 28 days and **(h)** 42 days after operation in experimental group. (black arrow in illustration (200×) represents material residue).

The H&E staining of the brain tissue wound revealed that at 7 days postoperatively, in the wound area of the experimental group, there was loose and edematous brain tissue, accompanied by minimal red blood cell infiltration, mild vascular congestion, and increased inflammatory cell infiltration. Some brain tissue exhibited liquefactive necrosis. Around the wound, a small number of fibroblasts and capillaries began to proliferate, initiating wound repair, and a very small amount of material residue was observed in the wound (Figure 12a). The edema and liquefactive necrosis of the brain tissue might have been related to the brain tissue damage caused by the surgical removal of part of the frontal lobe tissue. At 14 days postoperatively, mild local tissue edema persisted at the wound site in the experimental group. Numerous fine collagen fibers were observed covering the local surface, and there was still considerable inflammatory cell infiltration. Fibroblasts proliferated at the wound periphery, with the fibers became slightly thicker. A very small amount of material debris remained visible in the wound (see Figure 12b). At 28 days and 42 days postoperatively, the brain tissue morphology and structure of the animals in the sham operation group appeared clear and intact. A thin layer of fibrous connective tissue covered the meninges, and no hemorrhage, edema, or inflammatory infiltration were observed (Figures 12c,f). At 28 days postoperatively, both the experimental group and the marketed control group showed that a wound surface covered by thicker and mature granulation tissue. The tissue featured closely arranged collagen fibers, numerous capillaries, and a large number of fibroblasts. Local inflammatory cell infiltration was still considerable. In both groups, extremely small amounts of material residues persisted on the wound surfaces (Figures 12d,e). At 42 days postoperatively, both the experimental group and the marketed control group showed that the granulation tissue at the wound site was aging. There were dense arrangements of thick collagen fibers, a reduction in capillaries, more proliferation of fibroblasts and less infiltration of inflammatory cells. Moreover, in both the experimental group and the marketed control group, there were 2 animals each without any material residue, and there were 2 animals each with extremely small amounts of degraded residues (Figures 12g,h). It can be seen that the absorbable hemostatic powder basically completed its degradation and absorption 42 days after implantation. Its degradation and absorption process, as well as the tissue pathological reaction, were similar to those of the marketed control products.

## 4 Discussion

This work aims to investigate the hemostatic effect of absorbable hemostatic powder in a porcine model of intracranial and extracranial hemorrhage and evaluate its biological safety. The absorbable hemostatic powder used in this work was extracted from plant (potato) starch and prepared through emulsification technology to form a micro-porous polysaccharide (MPH) with a spatial network microstructure. The absorbable hemostatic powder has a porous surface with a porosity of up to 61.77%, and its water absorption rate exceeds 1,200%. Upon application to the wound, it can absorb water and low-molecular compounds from the blood to concentrate the solid components of the blood, and form a hemostatic gel matrix to accelerate hemostasis. A porcine intracranial hemorrhage model was used to evaluate hemostatic efficacy. The results demonstrated that the absorbable hemostatic powder achieved significant hemostasis, with a hemostasis time of 73.50 ± 29.08 s and a bleeding volume of 3.90 ± 2.09 g. These parameters were comparable to those of the marketed product (HaemoCer™), yet the required dosage was only half of that of HaemoCer™ hemostatic powder (*P* < 0.01). The biological safety of the absorbable hemostatic powder was evaluated based on the *in vitro* degradation studies and comprehensive animal testing, including clinical indicators, postoperative gross observation, and histopathological analysis. The results showed that the absorbable hemostatic powder degraded rapidly. The *in vitro* analysis revealed rapid degradation, with nearly complete breakdown within 48 h. After 42 days of implantation *in vivo*, the degradation and absorption processes were essentially complete. These findings indicate that the absorbable hemostatic powder possesses good biocompatibility, does not interfere with tissue healing, and helps prevent wound infection caused by minimizing foreign body reactions.

In conclusion, absorbable hemostatic powder was shown to be safe, reliable and easy to use. It performed effectively as an auxiliary hemostatic agent in neurosurgical models, aligning with key characteristics of new-generation absorbable neurosurgical hemostatic materials. These findings provide important guidance for future clinical application in neurosurgery and expected to further advance promotes the development of absorbable implantable hemostatic materials.

## Data Availability

The original contributions presented in the study are included in the article/supplementary material, further inquiries can be directed to the corresponding author.
